# Green Synthesis of Highly Fluorescent Carbon Dots from Bovine Serum Albumin for Linezolid Drug Delivery as Potential Wound Healing Biomaterial: Bio-Synergistic Approach, Antibacterial Activity, and In Vitro and Ex Vivo Evaluation

**DOI:** 10.3390/pharmaceutics15010234

**Published:** 2023-01-10

**Authors:** Dina Saeed Ghataty, Reham Ibrahim Amer, Mai A. Amer, Mohamed F. Abdel Rahman, Rehab Nabil Shamma

**Affiliations:** 1 Department of Pharmaceutics, Faculty of Pharmacy, October University for Modern Sciences and Arts (MSA), Giza 12451, Egypt; 2 Department of Pharmaceutics and Pharmaceutical Technology, Faculty of Pharmacy, Al-Azhar University, Cairo 11754, Egypt; 3 Department of Microbiology and Immunology, Faculty of Pharmacy, October University for Modern Sciences and Arts (MSA), Giza 12451, Egypt; 4 Department of Biology and Biochemistry, School of Life and Medical Sciences, University of Hertfordshire Hosted by Global Academic Foundation, Cairo 4813001, Egypt; 5 Department of Pharmaceutics and Industrial Pharmacy, Faculty of Pharmacy, Cairo University, Cairo 11562, Egypt

**Keywords:** carbon dots, hydrothermal treatment, bovine serum albumin, linezolid delivery, antibacterial, wound healing

## Abstract

A simple and green approach was developed to produce novel highly fluorescent bovine serum albumin carbon dots (BCDs) via facile one-step hydrothermal treatment, using bovine serum albumin as a precursor carbon source. Inherent blue photoluminescence of the synthesized BCDs provided a maximum photostability of 90.5 ± 1.2% and was characterized via TEM, FT-IR, XPS, XRD, UV-visible, and zeta potential analyses. By virtue of their extremely small size, intrinsic optical and photoluminescence properties, superior photostability, and useful non-covalent interactions with the synthetic oxazolidinone antibiotic linezolid (LNZ), BCDs were investigated as fluorescent nano-biocarriers for LNZ drug delivery. The release profile of LNZ from the drug delivery system (LNZ–BCDs) revealed a distinct biphasic release, which is beneficial for mollifying the lethal incidents associated with wound infection. The effective wound healing performance of the developed LNZ–BCDs were evaluated through various in vitro and ex vivo assays such as MTT, ex vivo hemolysis, in vitro antibacterial activity, in vitro skin-related enzyme inhibition, and scratch wound healing assays. The examination of LNZ–BCDs as an efficient wound healing biomaterial illustrated excellent biocompatibility and low cytotoxicity against normal human skin fibroblast (HSF) cell line, indicating distinct antibacterial activity against the most common wound infectious pathogens including *Staphylococcus aureus* (ATCC^®^ 25922) and methicillin-resistant *Staphylococcus aureus*, robust anti-elastase, anti-collagenase, and anti-tyrosinase activities, and enhanced cell proliferation and migration effect. The obtained results confirmed the feasibility of using the newly designed fluorescent LNZ–BCDs nano-bioconjugate as a unique antibacterial biomaterial for effective wound healing and tissue regeneration. Besides, the greenly synthesized BCDs could be considered as a great potential substitute for toxic nanoparticles in biomedical applications due to their biocompatibility and intense fluorescence characteristics and in pharmaceutical industries as promising drug delivery nano-biocarriers for effective wound healing applications.

## 1. Introduction

The total global number of skin injury cases has substantial healthcare ramifications, as it represents almost 50% of the world’s yearly expenditure in the aspect of healthcare [1]. Understandably, wound healing is well-known to be one of the most critical challenges in medical society [2]. As bacteria come in contact with the exposed skin surface area, they readily colonize in the exudative wound fluid and necrotic tissues, hence resulting in mounted inflammation and delayed wound healing [3]. The healing process of an infected wound is complicated, as the bacterial infection at the wound surface represents a major threat to public health in wound care management, which may transform normal wound healing into a chronic wound [4]. The process of healing skin wounds consists of a series of four successive phases comprising hemostasis, inflammation, cell proliferation, and tissue remodeling [2].

A wide range of advancements in biomaterials and nanomaterials-based regenerative approaches have been used to facilitate and accelerate wound healing via promoting tissue regeneration and executing an effective barrier against wound infection [2,5]. Over the last decade, carbon-based nanomaterials have been extensively used for their excellent antibacterial properties, wound healing, and sterilization applications, in addition to their great biocompatibility [5]. Among them, carbon dots (CDs), as a novel zero-dimensional (0D) nanomaterial in the carbon family, have attracted enormous interest owing to their extremely small size (<10 nm), easy surface functionalization, low toxicity, superior biocompatibility, high stability, obvious advantages in aqueous solubility, high surface area, and being eco-friendly [6,7]. Furthermore, it is extensively acknowledged that CDs exhibit unique optical, electrochemical, and fluorescent properties. As per these unique properties, CDs have displayed a promising potential use as a novel carbon nanostructure in many fields of biomedical applications for in vitro and in vivo bioimaging, wound dressing, and gene and drug delivery [5,8]. Besides, researchers reported that CDs possess intense photoluminescence following two-photon fluorescence excitation near the infrared region, hence fostering their applications in anti-stoke biosensing as well as bioimaging [8,9]. There are numerous studies as well about utilizing CDs as substrates in the structure of biosensor probes due to the CDs’ high surface area, hence possibly enhancing the electrochemical signal of electrodes and presenting further active sites for aptamer immobilization [10]. In addition, CDs have displayed great capabilities to be utilized as a fluorescent imaging probe in complex biological systems. Thus their distinct optical characteristics, biocompatibility, as well as low toxicity make them a potential candidate for in vivo applications [8].

Synthetic approaches that have been developed to synthesize CDs can be divided into top-down and bottom-up techniques. The top-down technique utilizes physical and chemical approaches starting from a vast range of either natural or chemical precursors, which assemble to synthesize CDs, including pyrolysis, microwave irradiation, hydrothermal method, and ultrasonic treatment. Whereas the bottom-up technique utilizes physical approaches only to nano-fragment inorganic larger carbon precursors, including graphene, graphite, and carbon nanotubes, such as electrochemical synthesis, laser ablation, and arc discharge [11]. Among these techniques, the hydrothermal method has drawn tremendous attention as it is cost-effective, fast, facile, and a green one-step method with decreased processing severity which uses deionized (DI) water only as a solvent [12,13]. It is worth highlighting that it has been the most commonly applied method since it is convenient and efficient [14]. Notably, no surface passivation agent is required, whereas only the carbon source plays an important role in the synthesis of the CDs [13,15]. 

Recently tremendous attention has also been directed toward green chemistry for the development of CDs derived from crude natural materials, natural polymers, and cost-effective and biodegradable materials [11]. CDs derived from crude natural materials, including biomass waste, are green and generally better than their chemical counterparts because of the renewable recourses and lack of chemicals involved and have been synthesized via hydrothermal techniques from several precursors, including peach gum, watermelon peels, strawberries, papaya, peanut shells, spent tea, and olive pits [11,16]. Not only was biomass waste utilized to develop CDs, the use of several natural polymers, including polysaccharides and proteins to generate CDs has attracted great attention due to their inherent biocompatibility, biodegradability, and distinct physicochemical and biological properties. Natural polymer-derived CDs have been synthesized via hydrothermal techniques from many precursors, including chitosan, gelatin, lignin, xylan, and silk fibroin [16]. Among the precursors recently examined across literature, it can be inferred that proteins are potential precursors to synthesize CDs and are interesting materials due to their enhanced adsorption capacity, low toxicity, superior biodegradability, and exquisite non-immunogenicity [12,17]. The conjugation mechanism of proteins with drug molecules has shed light on various research areas and stipulated new prospects for the development of novel drugs [18]. Bovine serum albumin (BSA), a model globular protein, is a biocompatible, biodegradable, highly aqueous-soluble, inexpensive, and non-toxic carrier, which conjugates with drugs through non-covalent interactions and presents a 76% sequence homology with the three-dimensional (3D) structure of human serum albumin (HSA) [18,19,20]. Furthermore, BSA facilitates the drug to be delivered and transported in the body and be easily given up at the surface of the cell. The flexibility in conformation, as well as the existence of reactive groups on BSA, facilitate its attachment with other substances of different structures [20,21]. BSA nanoparticles were reported to be able to control the drug encapsulation and release as well as extend its circulation in the bloodstream [20]. Additionally, as reported earlier, BSA as a protein source is advantageous for the complex wound healing process, whilst BSA-based biomaterials have displayed remarkable cell affinity intrinsic characteristics [21]. 

Linezolid (LNZ) has been one of the most promising topical oxazolidinone antibiotics for treating and avoiding abundant bacterial skin infections caused by injury, abrasion, and surgery. It is a Food and Drug Administration (FDA) approved drug for treating common wound infectious bacteria such as penicillin-resistant *Streptococcus pneumonia*, methicillin-resistant *Staphylococcus aureus* (MRSA), vancomycin-resistant *Enterococci* (VRE), and glycopeptide intermediate *Staphylococcus aureus* (GISA). LNZ exerts its antibacterial activity via inhibiting the protein synthesis of bacteria through attaching to the 23S portion of the 50S ribosomal subunit, hence hindering bacterial growth. LNZ, as a topical wound healing biomaterial, treats several wounds due to burns and diabetic foot ulcers, attributed to its effectiveness [2,22].

In this study, novel highly fluorescent antibacterial bovine serum albumin CDs (BCDs) were synthesized via cost-effective and green one-step hydrothermal treatment using BSA as the precursor carbon source. The obtained BCDs were thoroughly characterized using numerous microscopic, optical, and spectroscopic techniques, including transmission electron microscopy (TEM), Fourier-transform infrared spectroscopy (FT-IR), X-ray photoelectron spectroscopy (XPS), X-ray diffraction (XRD), UV-visible, as well as photoluminescence and zeta potential measurements. Then, the BCDs were loaded with various ratios of LNZ to prepare LNZ–BCDs nano-bioconjugate complexes as efficient drug delivery nano-systems. The developed LNZ–BCDs nano-bioconjugates exhibiting promising spectroscopic, in vitro drug release and ex vivo characteristics are expected to facilitate wound healing via a bio-synergistic approach. Finally, the biocompatibility, cytotoxicity, and overall wound healing performance of the developed LNZ–BCDs nano-bioconjugate were further investigated by evaluating the survival rate, anti-elastase, anti-collagenase, and anti-tyrosinase activities, and effective wound healing properties against normal human skin fibroblast (HSF) cell line via MTT, ex vivo hemolysis, in vitro antibacterial activity by broth microdilution method, in vitro skin-related enzyme inhibition as well as scratch wound healing assays.

## 2. Materials

Linezolid was generously provided by Pfizer, Egypt. Bovine serum albumin (BSA), ethylenediamine tetraacetic acid (EDTA), MTT (3-(4,5-dimethylthiazol-2-yl)-2,5-diphenyltetrazolium bromide) solution, N-2-hydroxyethylpiperazine-N-2-ethane sulfonic acid (HEPES) buffer, citrate buffer, 2-furanacryloyl-l-leucylglycyl-l-prolyl-l-alanine, kojic acid, Dulbecco’s modified Eagle’s medium (DMEM), fetal bovine serum–Dulbecco’s modified Eagle’s medium (FBS-DMEM), *N*-Methoxysuccinyl-Ala-Ala-Pro-chloromethyl ketone, and *N*-Methoxysuccinyl-Ala-Ala-Pro-Val-*p*-nitroanilide were all purchased from Sigma-Aldrich Co., St. Louis, MO, USA. *Clostridium histolyticum* (1 mg/mL) buffer and Dimethyl sulfoxide (DMSO) were obtained from Fisher Scientific, Loughborough, UK. Human red blood cells (HRBCs), normal human skin fibroblast (HSF) cell line, human leukocyte elastase, and mushroom tyrosinase were acquired from Nawah Scientific Inc., Cairo, Egypt. Deionized water (DI) was utilized in all the experiments. Analytical grade chemicals were used.

### 2.1. Methods

#### Synthesis of BCDs Nano-Biocarrier and LNZ–BCDs Nano-Bioconjugate

The fluorescent carbon dots (CDs) were typically synthesized via a green facile one-pot hydrothermal technique adopted by Amin et al. [23], with slight modification. Bovine serum albumin (BSA) was employed as a carbon source precursor for the development of the fluorescent bovine serum albumin carbon dots (BCDs) nano-biocarrier. Briefly, 3.6 g of BSA was dissolved in 80 mL deionized (DI) water under gentle stirring using a magnetic stirrer for 20 min until a clear solution was attained. The solution was then transferred into a 150 mL Teflon-lined hydrothermal stainless-steel autoclave reactor (OLT-150ML, OLLITAL Technology Co., Ltd., Xiamen, China) and then heated in an electrical oven at 200 °C for 6 h. After cooling down at room temperature, the resultant yellowish-brown solution was ultra-centrifuged using a 3-16KL centrifuge (Sigma, Germany) for 20 min at 15,000 rpm at 25 °C to remove large insoluble carbonaceous particles and was then filtered using a 0.22 μm membrane filter (Membrane Solutions^®^ sterile syringe filter, Auburn, WA, USA). For further purification, the transparent filtrate was then dialyzed in the dark using a dialysis membrane (1000 Da molecular weight cut off, Frey Scientific, Nashua, NH, USA) against deionized water for 48 h, and then the final fluorescent BCDs nano-biocarrier solution was obtained and stored in a dark, cool place for further analysis.

For the conjugation of LNZ on BCDs nano-biocarrier, aqueous solutions of LNZ (0.6 mg/mL) and BCDs nano-biocarriers were mixed at different ratios (BCDs: LNZ = 4:1, 2:1, 1:1, 1:3, and 1:5) under continuous stirring using a magnetic stirrer at 25 °C for 48 h in a dark condition. Finally, the resultant LNZ–BCDs nano-bioconjugates were placed in an amber bottle and stored in a dark, cool place for further studies.

### 2.2. Characterization of BCDs Nano-Biocarrier and LNZ–BCDs Nano-Bioconjugate

The transmission electron microscope (TEM) image of BCDs nano-biocarrier was captured by a JEM-2100 microscope (JEOL Ltd., Japan) operating at an acceleration voltage of 200 kV. Particle size analysis was conducted by Image J software. Fourier-transform infrared (FT-IR) spectra of the LNZ molecules, BSA, as well as lyophilized samples of BCDs nano-biocarrier, and LNZ–BCDs nano-bioconjugate, were recorded on an IRAffinity-1S FT-IR spectrometer (SHIMADZU Corporation, Kyoto, Japan) to analyze the surface functional groups. X-ray photoelectron spectroscopy (XPS) was performed to investigate the elemental composition of BCDs nano-biocarrier using K-ALPHA X-ray photoelectron spectrometer (ThermoFisher Scientific, Waltham, MA, USA) with Al-K*_α_* radiation. The crystal structure of BCDs nano-biocarrier was investigated via Bruker D8 DISCOVER X-ray diffractometer (XRD, Berlin, Germany) with Ni-filtered Cu-K*_α_* radiation (45 kV, 40 mA, and *λ* = 0.15416 nm). The optical properties of LNZ, BCDs nano-biocarrier, and LNZ–BCDs nano-bioconjugate were analyzed using UV-1800 240V UV spectrophotometer (SHIMADZU Corporation, Kyoto, Japan). A Lumina LF1201005 fluorescence spectrometer (ThermoFisher Scientific, Waltham, MA, USA) was used to conduct all the fluorescence measurements of the BCDs nano-biocarrier. Excitation, as well as emission slits, were maintained at 10 nm. Finally, zeta (ζ) potential measurements were performed to determine the surface charge of LNZ, BCDs nano-biocarrier, and LNZ–BCDs nano-bioconjugate using a Nano ZS90 zeta-sizer (Malvern, Worcestershire, UK) at room temperature. 

### 2.3. Drug Loading and Encapsulation Efficiency of LNZ–BCDs Nano-Bioconjugates

The drug loading efficiency (DLE) and drug encapsulation efficiency (DEE) of the prepared LNZ–BCDs nano-bioconjugates were estimated as per the method described by Jalali et al. [24]. Briefly, 5 mL of the LNZ–BCDs nano-bioconjugate was dialyzed in the dark against deionized water at 25 ± 0.2 °C for 48 h [25]. Afterward, the amount of free unbound LNZ drug molecules in the supernatant was quantified with UV-visible spectroscopy (SHIMADZU Corporation, Model UV-1800 240V, Japan) at 251 nm [26]. The DLE (%) and DEE (%) were estimated using the following equations:

(1) $$DLE\;\left(\%\right)=\frac{Weight\;of\;drug\;loaded\;in\;LNZ–BCDs\;nano–bioconjugate\;}{Total\;weight\;of\;LNZ–BCDs\;nano–bioconjugate}\times 100$$

(2) $$DEE\;\left(\%\right)=\frac{Weight\;of\;drug\;loaded\;in\;LNZ–BCDs\;nano–bioconjugate}{Initial\;weight\;of\;drug}\times 100$$

The experiment was performed in triplicate. The mean as well as standard deviation were recorded. 

### 2.4. In Vitro Drug Release Studies of LNZ from the LNZ–BCDs Nano-Bioconjugate

The in vitro release studies of LNZ from the LNZ–BCDs nano-bioconjugate and the free LNZ drug solution were conducted using the dialysis bag diffusion method, adopted by Singh et al. [27] with slight modifications. The dissolution medium was phosphate buffer saline (PBS) (pH 7.4), which mimics physiological pH. Afterward, 3 mL of the LNZ–BCDs nano-bioconjugate and the free LNZ drug solution were placed in dialysis bags (1000 Da molecular weight cut off, Frey Scientific, Nashua, NH, USA), immersed in 80 mL PBS (pH 7.4) in separate glass vials, and placed in the Shaker Incubator apparatus (ZHICHENG ZHWY-2102C, Shanghai, China) at 100 rpm at 37 ± 0.3 °C to mimic the skin temperature. Aliquots of 3 mL were taken from the dissolution medium at predefined time points at 0.5, 1, 2, 3, 4, 5, 6, 7, 8, 10, 12, 24, 36, and finally at 48 h. The sink conditions were maintained by immediately replenishing the equivalent volume of fresh PBS (pH 7.4) after each aliquot withdrawal. The concentration of LNZ released in the aliquots withdrawn from the dissolution medium was quantified spectrophotometrically via a UV spectrophotometer (SHIMADZU Corporation, Model UV- 1800 240V, Kyoto, Japan) at 251 nm. The study was performed in triplicate and was graphically presented as the cumulative percentage of drug release versus time. Data from the in vitro drug release study was further analyzed using different kinetic models to determine the drug release pattern.

### 2.5. *Ex Vivo Hemolysis Assay*

Ex vivo hemolysis assay was conducted according to the method described by Huang et al. [28]. Freshly collected human red blood cells (HRBCs) were separated by centrifugation for 10 min at 2500 rpm and then washed three times and dispersed in 4 mL of sterile PBS (pH 7.4). Afterward, 200 μL of diluted HRBCs was added to 600 μL of sterile PBS (pH 7.4) solution with diverse concentrations (25–300 μg/mL) of the LNZ–BCDs nano-bioconjugate. Deionized water and sterile PBS (pH 7.4) were employed as positive and negative controls, respectively. All samples were then shaken and incubated at 37 °C for 2 h and then centrifuged again at 2500 rpm for 15 min. The supernatant was collected, and optical density was recorded by UV-1800 240V UV spectrophotometer (SHIMADZU Corporation, Kyoto, Japan) at 541 nm while using sterile PBS (pH 7.4) as a blank. The hemolysis percentage was estimated using the following equation:

(3) $$Hemolysis\;percentage\;\left(\%\right)=\frac{\;A_{s}-A_{n\;}}{\;A_{p}-A_{n}\;}\times 100$$

where *A_s_*, *A_n_*, and *A_p_* present the absorbances of the sample, negative, and positive controls, respectively. The assay was conducted in triplicate. The mean as well as standard deviation were recorded.

### 2.6. Cytotoxicity Assay

The cell cytotoxicity and biocompatibility of BCDs nano-biocarrier and LNZ–BCDs nano-bioconjugate were assessed via MTT (3-(4,5-dimethylthiazol-2-yl)-2,5-diphenyltetrazolium bromide) assay against the normal human skin fibroblast (HSF) cell line, which was obtained from Nawah Scientific Inc., Egypt. Briefly, HSF cell line suspension (100 μL/well) was seeded into a 96-well plate, cultured in Dulbecco’s modified Eagle’s medium (DMEM) containing 100 mg/mL streptomycin, 100 units/mL penicillin, and 10% of heat-inactivated fetal bovine serum (FBS), and afterward incubated at 37 °C for 24 h under a humidified atmosphere with 5% CO_2_. Afterward, the cells were treated with various concentrations of BCDs nano-biocarrier and LNZ–BCDs nano-bioconjugate (25–1000 μg/mL) and incubated for an additional 24 h. Subsequently, the culture medium was cast-off, and the cells were washed using sterile PBS (pH 7.4) three times. MTT reagent (20 μL of 5 mg/mL stock solution) was added into each well and then incubated at 37 °C for another 4 h to facilitate the formation of the violet-colored formazan. In each well, the formed crystals of formazan were thoroughly dissolved using 200 μL of dimethyl sulfoxide (DMSO). After shaking the resultant solutions for 10 min, the optical density (OD) of each solution at λ_max_ 570 nm was recorded using a multi-well FLUOstar^®^ Omega microplate reader (BMG LABTECH, Baden-Wurttemberg, Germany). Cell viability was expressed as the percentage of survival cells using (Equation (4)) and reported as the mean values of triplicate measurements [29].

(4) $$Cell\;viability\;\left(\%\right)=\frac{OD_{t}}{OD_{c}}\times 100$$

where *OD_t_* and *OD_c_* represent the optical densities of the treated and control samples, respectively. 

### 2.7. In Vitro Antibacterial Activity by Broth Microdilution Method

The minimum inhibitory concentration (MIC) of pure LNZ, BCDs, and their formulated conjugate (LNZ−BCDs) were tested against pathogens commonly causing wound infection, such as *Staphylococcus aureus* (ATCC^®^ 25922) and methicillin-resistant *Staphylococcus aureus* (MRSA) that was previously isolated from dental clinic surface. The MIC was determined by the broth microdilution method. Stock solutions of LNZ were prepared at a concentration of 300 µg/mL and then sterilized by filtration. Stock solutions were serially diluted (2-fold) in sterile Müeller Hinton broth (MHB) with a dilution range of 0.125 to 150 µg/mL. Overnight culture turbidity was adjusted equivalent to the 0.5 McFarland standard and then diluted to give a final concentration equivalent to 10^6^ CFU/mL [30]. One hundred microliters of the adjusted bacterial culture were added to an equal volume of the tested dilutions in a 96-well microtiter plate. The plates were then incubated at 37 °C for 24 h. The effect of the conjugation between LNZ and BCDs was determined by measuring the change in the MIC of the pure LNZ and unconjugated BCDs. The synergistic and antagonistic activity of this conjugation was reflected by a decrease or increase in the MIC values, respectively, compared to the unconjugated LNZ.

The MIC was recorded as the lowest concentration of an antimicrobial agent, which gave no visible growth. Isolates were classified as susceptible (≤4 µg/mL), or resistant (≥8 µg/mL) to LNZ according to the Clinical and Laboratory Standards Institute (CLSI) breakpoints [31]. The experiment was conducted in triplicate. The mean as well as standard deviation were recorded.

### 2.8. In Vitro Skin-Related Enzyme Inhibitory Assays

The anti-elastase, anti-collagenase, and anti-tyrosinase activities were assessed following the method reported by Mostafa et al. [32]. The pure LNZ, BCDs nano-biocarrier, and LNZ–BCDs nano-bioconjugate were tested for in vitro skin-related enzyme activity inhibition assays. For the anti-elastase assay, human leukocyte elastase (1 µg/mL) was incubated with N-2-hydroxyethylpiperazine-N-2-ethane sulfonic acid (HEPES) buffer (pH 7.5). Each of the tested samples or the standard inhibitor *N*-Methoxysuccinyl-Ala-Ala-Pro-chloromethyl ketone (1.4 mg/mL) was added into the 96-well plate for 20 min at 25 °C before the addition of 100 µL substrate of 1 mM *N*-Methoxysuccinyl-Ala-Ala-Pro-Val-*p*-nitroanilide. Afterward, the optical density was measured after 40 min of incubation at 405 nm against a blank well lacking the test sample/inhibitor. Various concentrations of the tested samples (25–300 µg/mL) were used.

For the anti-collagenase, collagenase type 1 from *Clostridium histolyticum* (1 mg/mL) buffer (pH 7.4) along with tested samples or standard inhibitor ethylenediamine tetraacetic acid (EDTA) (1.4 mg/mL) were incubated at 37 °C for 20 min prior to the addition of 100 µL of the substrate 2-furanacryloyl-l-leucylglycyl-l-prolyl-l-alanine (FALGPA). The mixture was then incubated at 37 °C for an additional 1 h before the addition of 200 µL of 2% ninhydrin in 200 mM citrate buffer (pH 5) and placed in a water bath for 5 min. Afterward, the solution was left to cool down completely. Afterwards 200 µL of 50% isopropanol was added, and the optical densities were analyzed at 540 nm. Various concentrations of the tested samples (25–300 µg/mL) were used.

For the anti-tyrosinase assay, mushroom tyrosinase (5600 units/mL) was incubated at 37 °C for 15 min along with tested samples or standard inhibitor kojic acid (1.4 mg/mL) before the addition of the substrate 1 mM L-DOPA. Afterward, the optical density of the formed dopachrome was analyzed at 475 nm. Various concentrations of the tested samples (75–500 µg/mL) were used. All tests were conducted in triplicate. The enzyme inhibition activity was expressed in percentage using the following equation:

(5) $$Enzyme\;inhibition\;activity\;\left(\%\right)=\frac{Optical\;density\;of\;control\;–\;Optical\;density\;of\;sample}{Optical\;density\;of\;control}\times 100\;$$

### 2.9. Scratch Wound Healing Assay

For the scratch wound healing assay, a normal human skin fibroblast (HSF) cell line, acquired from Nawah Scientific Inc. (Cairo, Egypt), was plated at a density of 2 × 10^5^/well on a coated 12-well plate and subsequently cultured in a 5% fetal bovine serum–Dulbecco’s modified Eagle’s medium (FBS-DMEM) at 37 °C and 5% CO_2_ [33]. Afterward, the horizontal scratches were performed in the confluent monolayer, and the 12-well plate was then extensively washed using PBS (pH 7.4). The control wells were refilled with a fresh medium, whereas the test wells were treated using a fresh media of BCDs nano-biocarrier and LNZ–BCDs nano-bioconjugate for a period of 48 h. Images were taken after 0, 24, and 48 h of the incubation period via an inverted microscope (Carl Zeiss, Jena, Germany). The 12-well plate was then incubated at 37 °C and 5% CO_2_ in-between time points. The obtained images were then analyzed via Mill ImageView software version 3.7. The assay was performed in triplicate. The width of the wound gap (mm) was estimated as the mean distance between the edges of performed scratches [34]. Whereas the rate of cell migration (*RM*) was estimated using the following equation:

(6) $$RM=\frac{\;(W_{i}-W_{f})\;}{t}\;$$

where, *W_i_* and *W_f_* present the mean initial and final width of the wound gap, respectively, and *t* presents the migration duration (h). Furthermore, the area of the wound was estimated via tracing cell-free area in the captured images via Fiji-ImageJ public domain software version 1.53 (Bethesda, MD, USA), and wound closure (%) was calculated using the following equation:

(7) $$Wound\;closure\;\left(\%\right)=\frac{\;(A_{t=0}-A_{t=\Delta t})\;}{A_{t=0}}\times 100$$

where, *A_t_*_=0_ presents the mean wound area measured instantly after the scratch was performed, at time zero. *A_t_*_=*Δt*_ presents the mean wound area measured some hours after the scratch was performed. Results are expressed as the mean and standard deviation of three readings.

### 2.10. Statistical Analysis

Statistical analysis of in vitro drug release kinetic models was carried out using one-way analysis of variance (ANOVA) followed by Tukey’s Multiple Comparison post hoc test; *p* ≤ 0.05. Moreover, in vitro skin-related enzyme inhibitory assays were carried out in triplicates, and results were expressed as mean ± standard deviation. A non-linear regression curve was used to calculate IC_50_. An unpaired *t*-test was used for statistical comparison between the two groups. *p* < 0.05 is considered statistically significant. Analyses were done using GraphPad Prism 6.01 (San Diego, CA, USA). 

## 3. Results and Discussion

### 3.1. Synthesis of BCDs Nano-Biocarrier and LNZ–BCDs Nano-Bioconjugate

In this study, highly fluorescent CDs derived from BSA for LNZ drug delivery were designed for effective wound healing treatment. Numerous techniques have been proposed for synthesizing CDs, among which the hydrothermal method is regarded as the best method, as it is green and offers large-scale as well as facile synthesis procedures. In this study, BCDs nano-biocarriers were synthesized via one-step hydrothermal carbonization of BSA at 200 °C, resulting in the formation of a dark yellowish-brown solution from a yellow-colored solution which provided the first sign of the successful formation of BCDs [35]. This method effectively breaks down the native three-dimensional (3D) structure of BSA and renders carboxyl and amine groups extensively exposed [17]. Purification and separation of BCDs nano-biocarriers by dialysis yielded a pale yellow-colored solution. Afterward, to investigate the potential of BCDs nano-biocarrier in the delivery of antibacterial agents for effective wound healing, LNZ was loaded onto the BCDs. 

### 3.2. Transmission Electron Microscopy (TEM) Analysis

**Figure 1:**
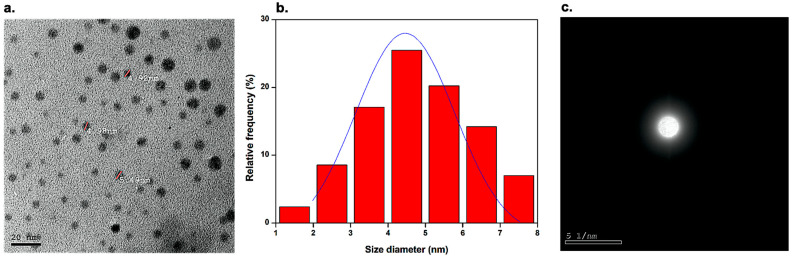
(**a**) Transmission electron microscope (TEM) image at the scale of 20 nm, (**b**) size distribution histogram, and (**c**) selected area electron diffraction (SAED) pattern of the BCDs nano-biocarrier.

TEM analysis was carried out to investigate the morphology of the synthesized BCDs nano-biocarrier. The TEM image of the BCDs nano-biocarrier is shown in Figure 1a. The BCDs nano-biocarrier clearly showed monodispersed spherical-like shape with a narrow size distribution and a relatively uniform size without any apparent aggregation. The statistical particle size distribution of the BCDs nano-biocarrier ranges from 1.5 ± 0.2 − 7.5 ± 0.1 nm with an average diameter of 4.5 ± 0.3 nm (Figure 1b). Selected area electron diffraction (SAED) pattern of BCDs nano-biocarrier presented diffused rings without any lattice spot or ring (Figure 1c). This corresponds to the amorphous nature of the graphitic carbon, which was further confirmed via XRD and XPS analyses [13,36]. Hence, it can be concluded that the synthesized BCDs nano-biocarrier is certainly within the quantum-sized materials [12].

### 3.3. Fourier-Transform Infrared Spectroscopy (FT-IR) Analysis

**Figure 2:**
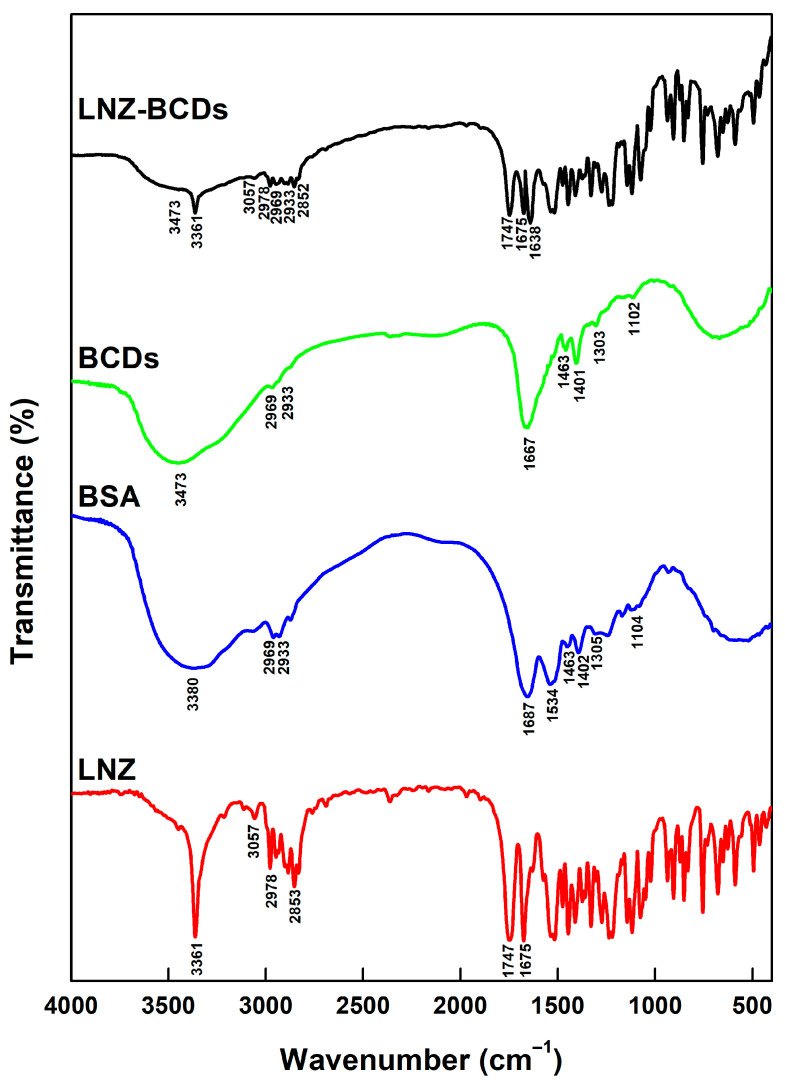
Fourier-transform infrared (FT-IR) spectra of the pure LNZ, pure BSA, BCDs nano-biocarrier, and LNZ–BCDs nano-bioconjugate.

FT-IR spectroscopy was performed to characterize surface functional groups and to confirm carbonization and conjugation processes. FT-IR spectra of pure LNZ, pure BSA, BCDs nano-biocarrier, and LNZ–BCDs nano-bioconjugate have revealed several typical characteristic bands, as presented in Figure 2 [15,17]. FT-IR spectrum of pure LNZ displayed distinctive bands at 3361 cm^−1^ for –NH group, 3057 cm^−1^ for aromatic –CH, 2978 and 2853 cm^−1^ for aliphatic –CH, and 1747 and 1675 cm^−1^ for C=O of an inner ester of five-membered ring and amide, respectively. Previous studies reported a similar FT-IR spectrum for LNZ pure drug [2,37].

At first, the FT-IR spectrum of pure BSA presented complex fingerprint bands attributed to the characteristic chemical structure of amino acids [17]. However, after the hydrothermal carbonization process, the FT-IR spectrum of BCDs nano-biocarrier became simpler and the absorption bands were slightly shifted due to the altered chemical environment (Figure 2). Many characteristic bands were still found in the FT-IR spectrum of BCDs nano-biocarrier, which correspond to –OH/–NH broadband at 3600–3299 cm^−1^, aliphatic –CH stretching at 2969 and 2933 cm^−1^, carbonyl (C=O) stretching at 1667 cm^−1^, C=C stretching at 1463 cm^−1^, C–N stretching at 1401 cm^−1^, C–O stretching at 1303 cm^−1^, and symmetric stretching vibration of C–O–C at 1102 cm^−1^ [38]. The absorption bands of both the carbonyl (C=O) stretching and the C–O stretching together indicate the existence of the –COO– functional group [39]. Moreover, a blue shift for the carbonyl (C=O) stretching was observed. This tendency can be attributed to the electron cloud existing between carbons; thus, oxygen atoms tend to move toward the direction of oxygen [40]. Other researchers reported similar findings for other CDs [15,39,40]. Additionally, the existence of the hydroxyl (−OH) functional group plays an important role in enhancing the antibacterial activity of BCDs nano-biocarrier, thus facilitating wound healing. Alike finding was previously reported by Pandiyan et al. [41] for CDs derived from sugarcane industrial wastes. 

After capping LNZ on the surface of the BCDs nano-biocarrier, the FT-IR spectrum of LNZ–BCDs nano-bioconjugate exhibited not only the distinctive bands of BCDs nano-biocarrier, but also the distinctive bands of LNZ (Figure 2). This observation confirmed the existence of the LNZ drug molecules in the product structure. Similar findings were obtained by Arsalani et al. [42] for the polyethylene glycol passivated CDs loaded with methotrexate. Nevertheless, the carbonyl (C=O) stretching in the LNZ–BCDs nano-bioconjugate spectrum was shifted to a lower wavelength (1638 cm^−1^). Ultimately, such reduction in the vibrational wavelength indicates the potential formation of an amide group and the successful tagging of LNZ with BCDs nano-biocarrier via non-covalent interactions [43,44]. Similar findings were previously reported by Hassan et al. [43] for ibuprofen grafted on nitrogen-doped CDs. The above-mentioned results indicate that the BCDs nano-biocarrier’s surface was enriched with plentiful hydroxyl, amino, and carboxyl groups. These functional groups can potentially act as active sites for attaching extrinsic molecules through either hydrogen bonding or van der Waals interactions. Therefore, the –NH and the two C=O groups in the LNZ drug molecules tend to form hydrogen bonds with the surface functional groups of BCDs nano-biocarrier. Similar results were previously reported by Shao et al. [39] for lycorine-loaded CDs. 

### 3.4. X-ray Photoelectron Spectroscopy (XPS)

**Figure 3:**
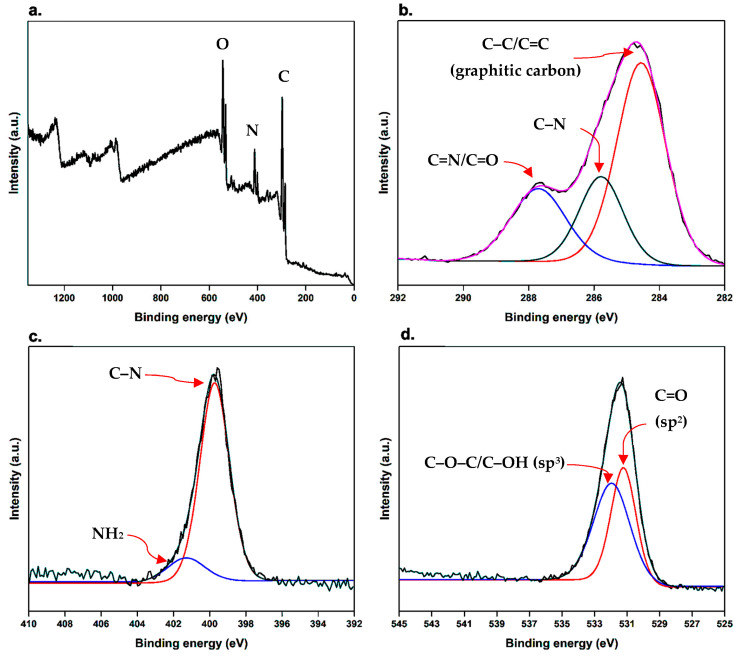
X-ray photoelectron (XPS) spectra of BCDs nano-biocarrier. (**a**) survey XPS spectrum and high-resolution spectra of (**b**) C1s, (**c**) N1s, and (**d**) O1s.

XPS was conducted to examine the surface elemental and chemical composition, as well as the nature of bonds existing in the synthesized BCDs nano-biocarrier, as shown in Figure 3. The total XPS survey spectrum indicated the presence of three major elements of C, N, and O, with atomic percentages of 64.36, 12.76, and 22.88%, respectively [36]. The distinct binding energy peaks observed at 285.76, 400.14, and 532.03 eV correspond to C1s, N1s, and O1s, respectively (Figure 3a). 

As demonstrated in the high-resolution XPS spectrum of C1s (Figure 3b), three main peaks were observed at 284.56, 285.79, and 287.70 eV, which correspond to C–C/C=C (for graphitic carbon), C–N, and C=N/C=O, respectively [15,36]. Similarly, the high-resolution spectrum of N1s (Figure 3c) can be deconvoluted in the presence of two characteristic peaks attributed to C–N at 399.73 eV and NH_2_ at 401.30 eV [15]. High-resolution O1s spectrum (Figure 3d) revealed two distinct binding energy peaks at 531.21 and 531.93 eV ascribing to C=O (sp^2^) and C–O–C/C–OH (sp^3^), respectively [39]. Interestingly, the results displayed that the surface of the synthesized BCDs nano-biocarrier may be provided with hydroxyl, amide, and carbonyl functional groups. Therefore, these surface hydrophilic functional groups not only remarkably enhance the stability and solubility of BCDs nano-biocarrier in the aqueous medium but also significantly foster applications in medicine and biology [15]. These results correlate well with the results of FT-IR. 

### 3.5. X-ray Diffraction (XRD)

**Figure 4:**
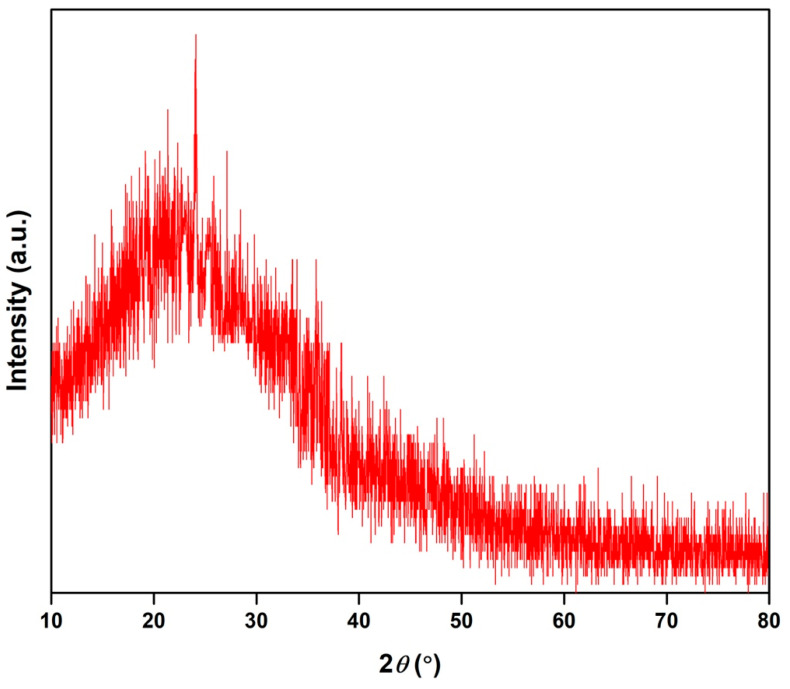
X-ray diffraction (XRD) pattern of BCDs nano-biocarrier.

The XRD was employed to investigate the crystalline nature of BCDs nano-biocarrier. As shown in Figure 4, the XRD pattern reveals a distinct broad diffraction peak located at 2*θ* = 24.01°, corresponding to interlayer spacing in graphite (0.34 nm) [15]. This finding indicates the amorphous carbon phase of BCDs nano-biocarrier without any crystalline phases and could attribute to the introduction of more oxygen-containing groups [1,45]. Furthermore, the results confirm that the BCDs nano-biocarrier exhibit graphitic nature with highly disordered carbon atoms, hence containing graphite-like structures [14,46]. These results are in good agreement with the data of XPS. Similar findings were reported by Rahmani et al. [47] and Hu et al. [15] for nitrogen-doped CDs and albumin CDs, respectively. 

### 3.6. UV-Visible Spectroscopy

**Figure 5:**
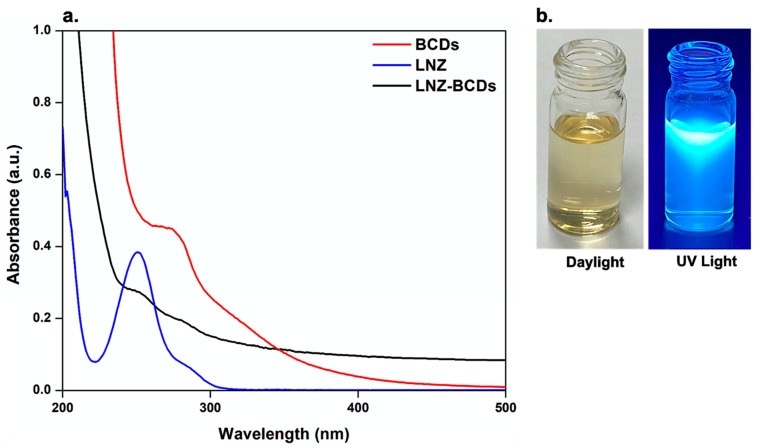
(**a**) UV-visible absorption spectra of pure LNZ, BCDs nano-biocarrier, and LNZ–BCDs nano-bioconjugate, and (**b**) photographic images of BCDs nano-biocarrier in daylight and under UV light excitation at 365 nm.

The optical properties of the pure LNZ, BCDs nano-biocarrier, and LNZ–BCDs nano-bioconjugate were characterized via analyzing their UV–visible absorption spectra. As shown in Figure 5a, the UV–visible absorption spectrum of pure LNZ exhibits a strong absorbance peak located at 251 nm, indicating the λ_max_ of the pure drug molecules [2]. It can be obviously seen that the UV-visible absorption spectrum of BCDs nano-biocarrier displays a distinct absorbance peak centered at about 280 nm (Figure 5a). This absorbance peak typically corresponds to π–π* electronic transition of C=C bonds, hence indicating the existence of aromatic π orbitals in the BCDs nano-biocarrier and corresponding to the characteristic feature of the protein α-helical structure [20,48]. A similar finding was reported by Amin et al. [23] for nitrogen-doped CDs using date kernel. Visually, it can be easily seen that the aqueous solution of BCDs nano-biocarrier shows a transparent, uniform, and pale-yellow colored solution in daylight and emits an obvious intense bright blue fluorescence under the UV light excitation at 365 nm (Figure 5b). This indicates the formation of BCDs nano-biocarrier exhibiting a relatively high quantum yield, similar to previously reported studies [12,46]. On the other hand, LNZ–BCDs nano-bioconjugate’s UV–visible absorption spectrum revealed that the absorbance peaks of both LNZ and BCDs nano-biocarrier were retained at 251 nm and 280 nm, respectively, thus confirming successful conjugation and attachment of LNZ drug molecules on the surface of BCDs nano-biocarrier. A similar finding was reported by Sun et al. [7] for the doxorubicin entrapped CDs. 

### 3.7. Fluorescence Spectroscopy

The fluorescence properties of the BCDs nano-biocarrier were investigated using fluorescence spectroscopy. It is worth noting that the BSA chain by itself is regarded as a fluorescent molecule, owing to the presence of two different types of tryptophan (Trp–134 and Trp–213) residues localized on the surface of the protein in domain I and in the hydrophobic cavity of domain II, respectively, which can interact or attach to other molecules [49]. 

**Figure 6:**
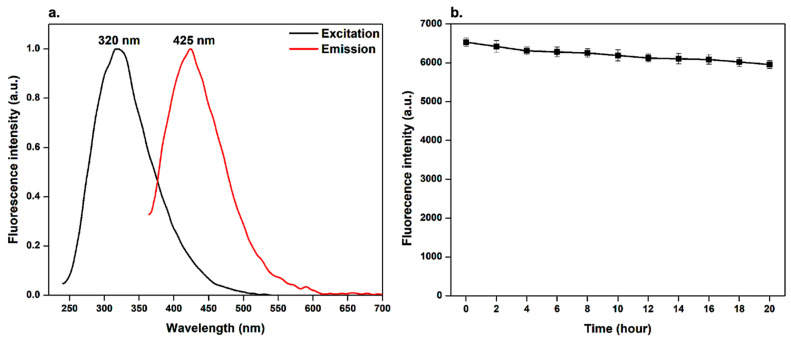
Fluorescence measurements for BCDs nano-biocarrier. (**a**) Fluorescence maximum excitation (λ_excitation_ = 380 nm) and emission (λ_emission_ = 480 nm) spectra, and (**b**) fluorescence intensity upon continuous excitation with UV-lamp at wavelength 365 nm.

As shown in Figure 6a, the fluorescence excitation and emission spectra of the BCDs nano-biocarrier present a distinct maximum emission peak centered at about 425 ± 1.2 nm when excited at an optimal wavelength of around 320 ± 1 nm. This can be ascribed to the amino acid residues present in the BCDs nano-biocarrier, including tryptophan (Trp), tyrosine (Tyr), and phenylalanine (Phe) [50]. The mechanism of the fluorescence emission for BCDs nano-biocarrier is due to band gap-transition. This is reasonably attributed to the fact that the maximum excitation peak centered at 320 nm (Figure 6a) is so near to the absorbance peak of the BCDs nano-biocarrier at a wavelength around 280 nm (Figure 5a), as previously reported [49]. Interestingly, the functional groups (–COOH, –OH, and –NH_2_) present on the BCDs nano-biocarrier surface, which were previously confirmed via FT-IR analysis, can result in a series of emissive traps that is of pronounced importance for the strong fluorescent properties displayed by the BCDs nano-biocarrier [46]. Furthermore, the fluorescence emission spectrum of the BCDs nano-biocarrier exhibits an obvious good symmetry with a narrow full width at half maximum (FWHM) of 89 nm. This indicates a narrow size distribution and monodispersed BCDs nano-biocarrier [25,51]. Notably, these results correlate well with results obtained from the TEM analysis. Similar findings were reported by other researchers [15,52]. 

### 3.8. Photostability 

The photostability of BCDs nano-biocarrier was investigated upon continuous exposure to UV light irradiation for 20 h using Vilber VL-6 LC UV-lamp (France) at wavelength 365 nm [15,28]. It was observed that the fluorescence intensity of the BCDs nano-biocarrier displays no significant change in the intensity and still preserves around 90.5 ± 1.2% after 20 h, as presented in Figure 6b. This tendency confirms that the BCDs nano-biocarrier exhibit high photostability and great resistance to photobleaching, as well as providing a potential alternative chemical for bioimaging applications [28]. Similar findings were reported by other researchers [15,28].

### 3.9. Zeta Potential 

**Figure 7:**
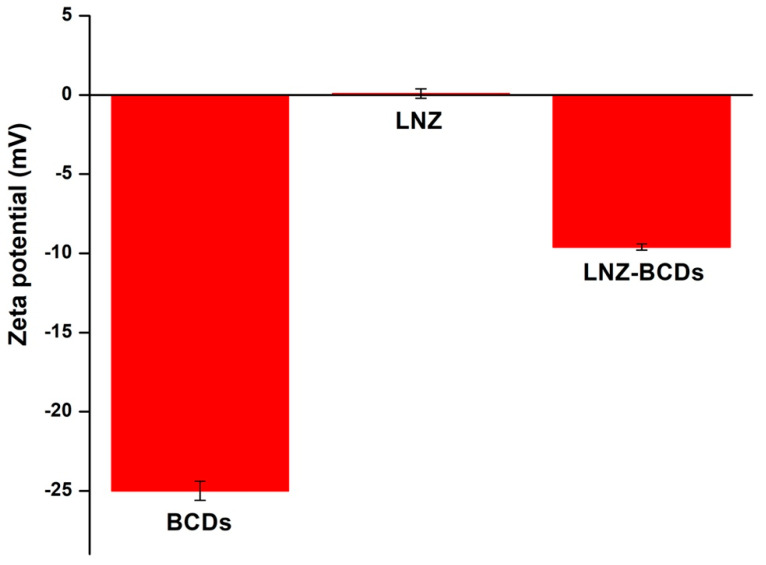
The ζ-potential measurements for BCDs nano-biocarrier, LNZ drug molecules, and LNZ–BCDs nano-bioconjugate.

The zeta (ζ) potential measurements were carried out to investigate the surface charge and assess the stability of the florescent BCDs nano-biocarrier against aggregation, as well as to confirm the conjugation of the BCDs nano-biocarrier with the synthetic antibiotic, LNZ [23]. The ζ-potential value of the BCDs nano-biocarrier was found to be −25.0 ± 0.7 mV (Figure 7), hence indicating good electrostatic stabilization ascribed by the high negative charge, which prevents agglomeration via strong inter-particulate electrostatic repulsion forces [20,40]. The high negatively charged surface of the BCDs nano-biocarrier can be attributed to the abundance of hydroxyl, carboxyl, and amino characteristic functional groups anchoring on the surface of BCDs nano-biocarrier, in consistency with the FT-IR and XPS data [13,42]. The remarkable electrostatic stability of BCDs nano-biocarrier confirms their convenience for several applications [53].

Interestingly, the physical conjugation of the electrically neutral LNZ on the surface of the negatively charged BCDs nano-biocarrier was demonstrated through a shift in ζ-potential value from −25.0 ± 0.7 mV to −9.6 ± 0.2 mV (Figure 7). This decrease in the surface charge confirmed the formation of LNZ–BCDs nano-bioconjugate complexes with a ζ-potential value of −9.6 ± 0.2 mV. This can be ascribed to the physical interactions between LNZ and BCDs nano-biocarrier via strong π–π interactions among the benzene structures, where the BCDs exhibit sp^2^-carbon network that can load the aromatic structure of LNZ [54]. Thus, LNZ presents a high affinity for the negatively charged BCDs nano-biocarrier. Similar findings were previously reported in the literature for other CDs [7,54].

### 3.10. Drug Loading and Encapsulation Efficiency of LNZ–BCDs Nano-Bioconjugates

The developed LNZ–BCDs nano-bioconjugates were designed to facilitate wound healing and promote tissue regeneration. LNZ, as a model synthetic oxazolidinone antibiotic with low aqueous solubility, was selected to study the drug loading efficiency (DLE) and drug encapsulation efficiency (DEE) of the BCDs nano-biocarrier [2]. As presented in Table 1, the DEE and DLE efficiency substantially increased as the ratio of BCDs:LNZ increased up to 1:1. DEE/DLE were 91.8 ± 1.3/20.7 ± 0.69, 92.5 ± 1.4/21.1 ± 0.19, and 97.2 ± 1.0/22.5 ± 0.23% when the ratio of BCDs:LNZ was 4:1, 2:1, and 1:1, respectively (Table 1). A similar result was reported for piperine-loaded BSA/oxidized gum Arabic nanocomposites in which DEE and DLE increased upon increasing the concentration of piperine [24]. Moreover, Rahmani et al. [47] prepared pH-responsive doxorubicin-loaded chitosan-montmorillonite-nitrogen-doped CDs for targeted treatment of cancer cells with high entrapment and loading efficiency of 91 and 49%, respectively. Higher ratios of BCDs:LNZ (1:3 and 1:5) were counterproductive, whereas the DEE/DLE declined to 88.1 ± 1.9/19.8 ± 0.36 and 85.3 ± 1.1/18.6 ± 0.47%, respectively. This was probably attributed to the drug precipitation [55]. Overall, the maximum DEE/DLE that could be obtained was 97.2 ± 1.0/22.5 ± 0.23% when the ratio of BCDs to LNZ was 1:1. Hence, this might be ascribed to the possible hydrogen bonding and π-π stacking interactions [20,45].

### 3.11. In Vitro Drug Release Studies of LNZ from the LNZ–BCDs Nano-Bioconjugate

**Figure 8:**
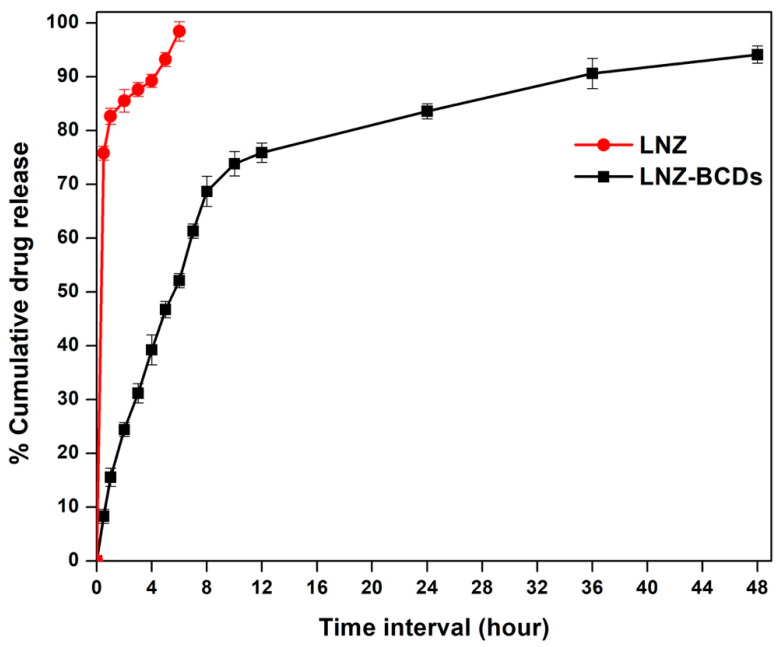
In vitro release profile of the LNZ–BCDs nano-bioconjugate in comparison to the LNZ drug solution in PBS (pH 7.4) at 37 ± 0.2 °C for up to 48 h.

An ideal wound healing biomaterial must postulate complete drug release over a long period of up to 24 to 48 h [2]. The cumulative release profile of the LNZ–BCDs nano-bioconjugate in comparison to the free LNZ drug solution was studied for 48 h, as shown in Figure 8. It was observed that the percentage LNZ released from the free drug solution was almost 98 ± 0.5% after 6 h. This resembles a fast and uncontrolled release manner. Nevertheless, the release of LNZ from the LNZ–BCDs nano-bioconjugate displayed a controlled release manner since the percentage of LNZ released after 6 h did not exceed 52 ± 1.3% [56]. The LNZ–BCDs nano-bioconjugate achieved almost complete drug release within 48 h in comparison to that of free LNZ drug solution within 6 h only (Figure 8). These findings indicate that LNZ–BCDs nano-bioconjugate significantly controlled LNZ release from the synthesized LNZ–BCDs nano-bioconjugate. 

The release profile of LNZ from the LNZ–BCDs nano-bioconjugate during the experiment displayed a biphasic release pattern, including a relatively fast-release property in the first 12 h, subsequently followed by a sustained release for a prolonged time for 48 h of the experimental period. A similar finding was reported by Karami et al. for isophethalaldoxime palladacycle complex-loaded BSA nanoparticles with a cumulative release profile over a period of 45 h in PBS pH 7.4 at 37 °C [20]. In the initial fast-release phase, almost 75 ± 1.8% of the drug was released from the LNZ–BCDs nano-bioconjugate within 12 h. This is due to the constant release rate of LNZ in the dissolution medium attributed to the weakly stacked drug molecules on the superficial zones of the graphene-like structures of the BCDs nano-biocarrier, which were controlled by a diffusion process due to the π–π interactions [20,54]. This behavior is first and foremost advantageous to exert the antibacterial action in the wound bed throughout the first few hours via drawing mesenchymal cells [2]. Subsequently, in the sustained LNZ release phase, the rate of LNZ release from the LNZ–BCDs nano-bioconjugate was gradual and much slower. This can be ascribed to the increasing diffusion pathways’ distances and to the remaining LNZ drug fraction entrapped in the inner core of the LNZ–BCDs nano-bioconjugate, which consistently diffused slowly from the matrix to the dissolution medium [20,25]. This tendency prevents recolonization and stimulates tissue renovation in the subsequent phases of the wound healing mechanism. Hence, this postulates a potentially mesmerizing opportunity for the synthesized LNZ–BCDs nano-bioconjugate for regenerative medicine. A similar finding was reported by Mehta et al. for lisinopril-loaded CDs derived from pasteurized milk with a cumulative release profile over 48 h in PBS pH 5.2, 6.2, and 7.4 at 37 °C [25]. 

An interesting point that is noteworthy to highlight is the influence of the negative ζ-potential value of the LNZ–BCDs nano-bioconjugate (−9.6 ± 0.2 mV) on the drug release profile. It was found that negative ζ-potential facilitates the drug delivery process and aids in wound healing via allowing the LNZ–BCDs nano-bioconjugate to easily penetrate through the cellular membrane and progressively increase the percentage LNZ released to accumulate in the cytoplasm and exert its therapeutic effect [53]. Of interest, the total percentage LNZ released from the LNZ–BCDs nano-bioconjugate by the end of the 48 h was approximately 95 ± 1.6%. This confirmed the proper loading efficiency of the LNZ drug molecules within the LNZ–BCDs nano-bioconjugate. This finding is in good correlation with previously reported studies [20,43].

Kinetic profile analysis for the LNZ–BCDs nano-bioconjugate release data was carried out according to zero order, first order, second order, Higuchi diffusion, Hixon-Crowel, and Baker-Lonsdal mathematical functions. To determine the drug release kinetics, cumulative release data of LNZ from LNZ–BCDs nano-bioconjugate were fitted in various models (Figure S1 and Table 2). According to the correlation coefficient (*R*^2^) value attained for each of the above-mentioned kinetic models, it was observed that the release of LNZ from the drug delivery system (LNZ–BCDs) seemed to be best fitting to the Second order model order kinetics as the plots revealed the prime linearity (*R*^2^ value = 0.985). 

### 3.12. Ex Vivo Hemolysis Assay 

**Figure 9:**
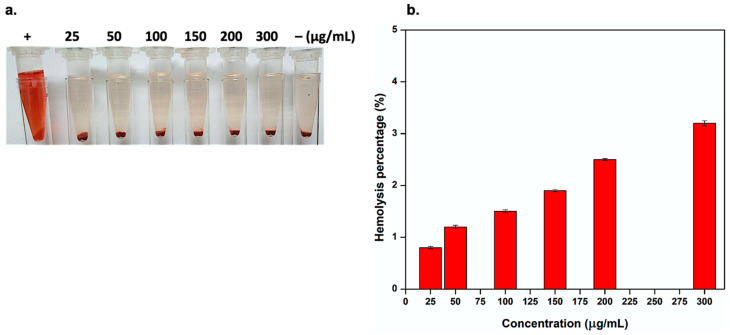
Hemolytic activity of LNZ–BCDs nano-bioconjugate. (**a**) Hemolytic images after treatment with LNZ–BCDs nano-bioconjugate of different concentrations (25–300 μg/mL). Particularly, (–) and (+) signs correspond to the negative and positive controls, respectively, and (**b**) hemolysis percentage induced by the LNZ–BCDs nano-bioconjugate.

The hemolysis assay was performed to further investigate ex vivo blood biocompatibility. It is one of the most important properties that should be evaluated for any biomaterial intended to be in contact with blood elements to prevent serious adverse effects for in vivo drug-delivery applications [57,58]. In the current study, the potential hemolytic activity induced by the synthesized LNZ–BCDs nano-bioconjugate wound healing biomaterial was investigated as the indication of ex vivo blood biocompatibility [58]. As shown in Figure 9a, the solution in deionized water was used as a positive control and turned red color due to hemolysis resulting in the release of hemoglobin (Hb) from the red blood cells (RBCs), whereas the negative control (PBS solutions) did not show any hemolysis [59]. The hemolytic images presented in Figure 9a show no obvious hemolysis in the PBS solutions with different LNZ–BCDs nano-bioconjugate concentrations (25–300 μg/mL). Although the hemolytic activity slightly increased at higher LNZ–BCDs nano-bioconjugate concentration, the hemolysis percentage was retained less than 5% even at concentrations up to 300 μg/mL (Figure 9b). It is worth highlighting that the LNZ–BCDs biomaterial is regarded as a non-toxic, non-hemolytic, and feasible blood-contacting material with favorable biocompatibility with RBCs, hence intended for secure in vivo drug-delivery applications, as the ISO 10993-4 precisely outlines safe biomaterials as having a hemolysis percentage ≤5% [57,60]. Of interest, it was previously reported that the hemolysis percentage of plain LNZ drug molecules was found to be 14% [59]. Hence, the less hemolysis of the LNZ–BCDs nano-bioconjugate overcame the high hemolysis percentage associated with plain LNZ drug molecules, which portrays their potential for drug delivery. Therefore, the results of the ex vivo hemolysis assay suggest that the LNZ–BCDs biomaterial is proper for medical application and potentially feasible for facilitating wound healing through blood circulation, similar to previously reported studies [58,61]. 

### 3.13. Cytotoxicity Assay

**Figure 10:**
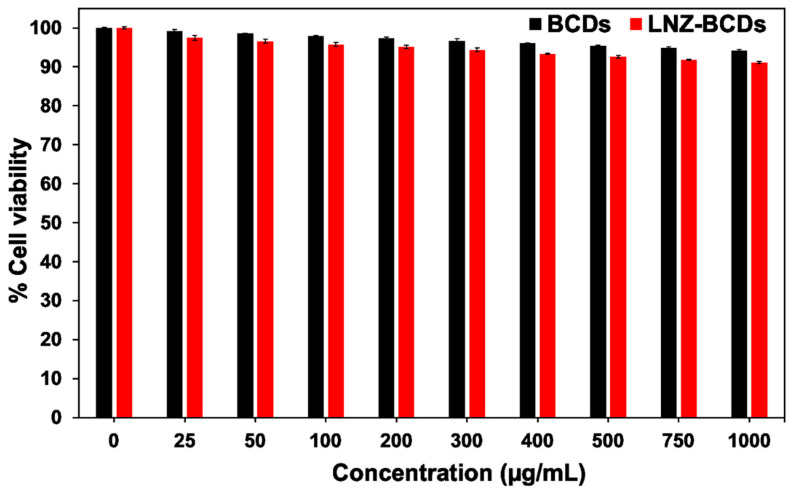
Cell viability percentage of HSF cell line against BCDs nano-biocarrier and LNZ−BCDs nano-bioconjugate at various concentrations for 24 h.

MTT assay was carried out against the HSF cell line to evaluate the biocompatibility and cytotoxicity of the synthesized BCDs and LNZ–BCDs nano-biomaterials. As shown in Figure 10, the cell viability of both BCDs and LNZ–BCDs nano-biomaterials displayed no noticeable toxicity to the HSF cell line after incubation for 24 h, confirming that these nano-biomaterials exhibit excellent biocompatibility. By comparing the cytotoxicity results of the BCDs nano-biocarrier with that of LNZ–BCDs nano-bioconjugate, the LNZ-loaded BCDs presented slightly higher cell cytotoxicity than the plain BCDs nano-biocarrier. However, the LNZ–BCDs nano-bioconjugate still maintained high cell viability above 91% even at a high concentration of 1000 µg/mL. This indicates that LNZ–BCDs nano-bioconjugate presented excellent biocompatibility and extremely low cytotoxicity and provides further proof as a promising potent drug delivery nano-system [7,42]. These findings postulate that LNZ–BCDs nano-bioconjugate could be an ideal candidate for escaping the dose-dependent side effects of the free LNZ, including gastrointestinal disorders, skin rashes, and allergic reactions, due to their relatively small mean particle size and shielding effects, and confirm the potential secure biomedical applications [40,42]. Similar studies [55,62] revealed that LNZ-loaded nano-systems exhibit higher antibacterial activity and lower cytotoxicity as compared with the conventional free LNZ, indicating their potential therapeutic effects.

### 3.14. In Vitro Antibacterial Activity by Broth Microdilution Method

The MIC of pure LNZ, BCDs nano-biocarrier, and their formulated nano-bioconjugate LNZ−BCDs was detected against MRSA and *Staphylococcus aureus* (ATCC^®^ 25922). Both isolates were sensitive to pure LNZ and their MIC was 4 µg/mL, as shown in Table 3. The BCDs nano-biocarrier showed a slight antibacterial activity against both MRSA and *Staphylococcus aureus* (ATCC^®^ 25922) (Table 3). This finding correlates well with the data mentioned in the FT-IR analysis, indicating that the existence of a hydroxyl (−OH) functional group in the structure of BCDs nano-biocarrier plays an important role in enhancing its antibacterial activity. Previous studies [63,64] have also shown that CDs exhibit antibacterial activity. 

Interestingly, the conjugation between LNZ drug molecules and BCDs nano-biocarrier in the formulated nano-bioconjugate LNZ−BCDs significantly reduced the MIC of LNZ and increased the bacterial sensitivity. The conjugation showed synergistic antibacterial activity subscribed to both LNZ and BCDs bio-nanocarrier together in the LNZ–BCDs nano-bioconjugate in comparison with their singular use, where the MIC of LNZ was distinctively reduced against *Staphylococcus aureus* (ATCC^®^ 25922) by 2-fold and against MRSA by 16-fold (Table 3). Therefore, it is worth highlighting that the nano-bioconjugated formula of both LNZ and BCDs nano-biocarrier possessed synergistic antibacterial activity and declined the growth of both MRSA and *Staphylococcus aureus* (ATCC^®^ 25922), which are major causes for wound infections. Furthermore, the formulated nano-bioconjugate LNZ−BCDs displayed even better antibacterial activity as the amino functional groups on the surface, positive surface charge, as well as small particle size facilitated the quick binding to the bacteria (negative charge on the surface), thus destroying the surface charge and eventually entering the bacteria [61]. Accordingly, the nano-bioconjugate LNZ−BCDs has the potential for use as a wound healing biomaterial to diminish bacterial load and avoid colonization at the wound bed, and hence facilitate the wound healing process.

### 3.15. In Vitro Skin-Related Enzyme Inhibitory Assays

The present study performed a screening of pure LNZ, BCDs nano-biocarrier, and LNZ–BCDs nano-bioconjugate regarding their in vitro inhibition of different skin-related enzymes, namely tyrosinase, collagenase, and elastase. The effect of pure LNZ, BCDs nano-biocarrier, and LNZ–BCDs nano-biomaterials on the percentage enzyme inhibition activity along with the IC_50_ (µg/mL) of various skin-related enzymes in comparison to their corresponding positive controls is presented in Table 4. 

Results revealed that LNZ–BCDs nano-bioconjugate had a significantly higher anti-elastase activity compared to each of LNZ (*p* = 0.006) and BCDs nano-biocarrier (*p* = 0.04) and a significantly higher anti-collagenase activity compared to LNZ (*p* < 0.0001) and positive control (*p* = 0.0017) (Table 4). Furthermore, LNZ–BCDs nano-bioconjugate illustrated a significantly higher anti-tyrosinase activity than LNZ, BCDs nano-biocarrier, and positive control (*p* < 0.0001, *p* = 0.0147, and *p* = 0.001, respectively). It is worth highlighting that the LNZ–BCDs nano-bioconjugate showed a significantly lower IC_50_ for the different skin-related enzymes than all other samples (all *p* < 0.01). The only exception was the anti-tyrosinase IC_50_ of BCDs nano-biocarrier, which was equal to the LNZ–BCDs nano-bioconjugate IC_50_ (*p* = 0.908) (Table 4). Therefore, the LNZ–BCDs nano-bioconjugate exhibited the highest and most robust anti-elastase, anti-collagenase, and anti-tyrosinase activities compared with pure LNZ and BCDs nano-biocarrier. These results indicate the synergistic effect subscribed to both LNZ and BCDs nano-biocarrier together in the LNZ–BCDs nano-bioconjugate in comparison with their singular use. 

The strong anti-elastase and anti-collagenase activities of LNZ–BCDs nano-bioconjugate potentially plays a vital role in re-epithelialization and shortening the duration of the wound healing process. Previous researchers [65] reported in the literature that elastase and collagenase may potentiate deprivation of the extracellular matrix and growth factors that prolong the inflammatory phase, thus delaying the wound healing process. Hence, preventing unnecessary elastase and collagenase at the wound site is relatively important. Moreover, the great anti-tyrosinase activity presented by LNZ–BCDs nano-bioconjugate would be beneficial in the treatment of hyperpigmentation observed during and after the wound healing process [66]. Hence, the LNZ–BCDs nano-bioconjugate showed a promising wound healing potential and a melanogenesis-inhibiting activity.

### 3.16. Scratch Wound Healing Assay

**Figure 11:**
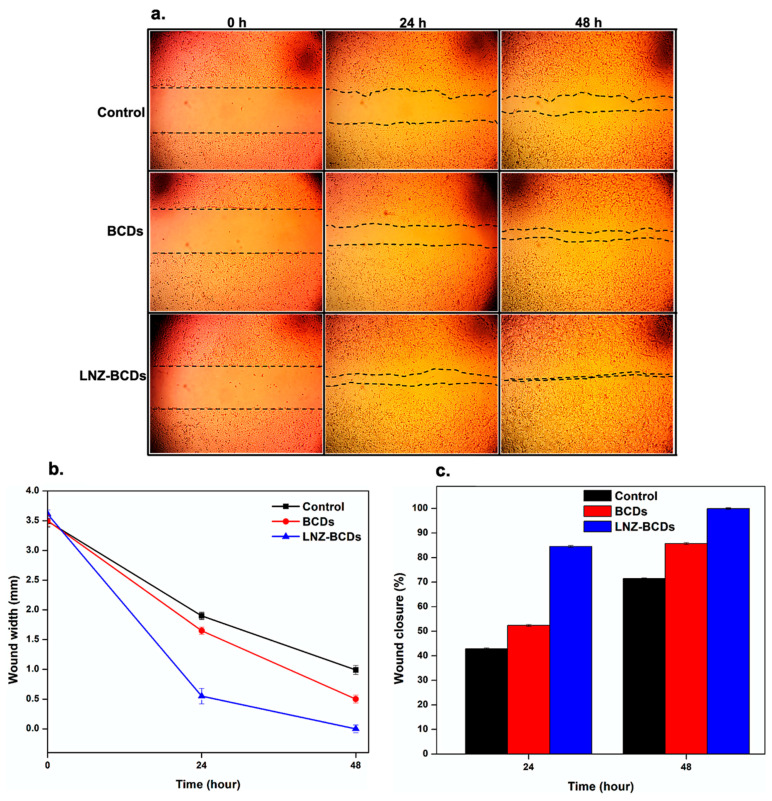
(**a**) Images at 1 mm scale bar, (**b**) profile of changes in the width of wounds with time, and (**c**) wound closure percentages of untreated control and treated normal human skin fibroblast (HSF) cell line with BCDs nano-biocarrier and LNZ–BCDs nano-bioconjugate at 24 and 48 h.

The evaluation of the effectiveness of BCDs and LNZ–BCDs nano-biomaterials in the wound healing process was determined in vitro using normal human skin fibroblast (HSF) cell line. In the current study, the scratch wound healing assay was selected as it is considered to mimic an incision wound in vitro [19]. The prime advantage of this assay is its relative feasibility and simplicity. The classic scratch wound primarily heals via the proliferation and migration of cells along with contraction and closure of the wound gap; thus, it is necessary to closely monitor the wound healing rate by imaging to confirm the complete wound healing process and tissue regeneration [34]. Changes in the width of wounds for treated and untreated HSF cell lines were assessed at 24 h and 48 h, and images are presented in Figure 11a,b. The wound closure percentages for treated and untreated HSF cell lines are graphically represented in Figure 11c.

During the assay, significant differences in the improvement of the wound status for the treated cells with BCDs and LNZ–BCDs nano-biomaterials from the untreated control cells were witnessed. Cells treated with BCDs nano-biocarrier illustrated considerably higher contraction and closure of the wound gap compared to untreated control cells throughout the assay. After 48 h, BCDs nano-biocarrier allowed faster wound closure than untreated control cells with approximately 85.75 ± 0.03% wound closure, while the wound width was reduced to 0.5 ± 0.04 mm of the initial wound width (3.50 ± 0.08 mm). On the other hand, cells treated with LNZ–BCDs nano-bioconjugate illustrated a significantly higher contraction and closure of the wound gap with approximately 84.53 ± 0.13% wound closure compared with both the untreated control and BCDs nano-biocarrier treated cells after 24 h. Wound closure analysis revealed complete closure of the scratch wound gap for the LNZ–BCDs nano-bioconjugate treated cells (99.97 ± 0.02%) compared with both untreated control (71.43 ± 0.02%) and BCDs nano-biocarrier treated (85.75 ± 0.03%) cells, which failed to heal completely after 48 h. These differences confirm the synergistic effect and excellent healing efficiency subscribed to both LNZ and BCDs nano-biocarrier together in the LNZ–BCDs nano-bioconjugate in comparison with the singular use of BCDs nano-biocarrier. Similar to the skin-related enzyme inhibition results, the LNZ–BCDs nano-bioconjugate combining both LNZ and BCDs nano-biocarrier exhibited robust anti-elastase, anti-collagenase, and anti-tyrosinase activities, thus accelerating wound healing and tissue regeneration processes. 

It is interesting to shed light on the rate of cell migration analysis, which illustrated that LNZ–BCDs nano-bioconjugate enabled a higher rate of cell migration of 75.21 ± 0.02 µm than other BCDs nano-biocarrier treated and untreated control cells of 62.50 ± 0.02 and 52.08 ± 0.03 µm, respectively, by 48 h. This indicates that the developed LNZ–BCDs nano-bioconjugate was capable of remarkably enhancing and promoting the fibroblasts proliferation and migration favoring the wound healing mechanism due to the presence of both LNZ and BCDs nano-biocarrier together as compared to BCDs nano-biocarrier treated and untreated control cells, which presents promising wound healing properties. These findings are consistent with the results of Rohiwal et al. [34] for bovine serum albumin (BSA)-dextran bio-nanoconjugate.

## 4. Conclusions

A highly fluorescent BCDs nano-biocarrier was successfully synthesized via green, facile, and one-step hydrothermal treatment without any further chemical modification. The developed BCDs nano-biocarrier, emitting bright blue fluorescence, illustrated extremely small size (1.5 ± 0.2–7.5 ± 0.1 nm), easy surface functionalization, intrinsic optical and fluorescence properties, high water solubility, superior photostability, and distinctive antibacterial activity. An innovative biphasic release BCDs of LNZ, which retains the intrinsic characteristics that meet the desirable properties of an ideal wound healing biomaterial, was successfully developed at an optimum ratio of 1:1, which showed a high drug encapsulation and loading efficiency of 97.2 ± 1.0% and 22.5 ± 0.23%, respectively. The developed LNZ–BCDs drug delivery nano-system did not impose any significant cytotoxicity with HSF cells and maintained high cell viability >91%, thus might be utilized as a potential candidate in many fields of biomedical applications. The in vitro and ex vivo results confirmed that the LNZ–BCDs nano-bioconjugate possessed excellent biocompatibility with HRBCs with hemolysis percentage <5%, distinct antibacterial activity against the most common wound infectious pathogens, including *Staphylococcus aureus* (ATCC^®^ 25922) and methicillin-resistant *Staphylococcus aureus,* robust anti-elastase, anti-collagenase, and anti-tyrosinase activities, and an overall promising wound healing performance with accelerated wound closure and improved tissue regeneration. To our knowledge, this is the first reported study to synthesize highly fluorescent CDs derived from BSA for LNZ drug delivery, which displays great potential for future in vivo and clinical applications. Given the results of this study, it can be speculated that the synthesized novel LNZ–BCDs drug delivery nano-system could bring a paradigm shift in the use of biomaterials for biomedical applications such as biosensing, bioimaging, photocatalysis, and for potential theranostic applications as a promising agent that synchronously recognizes, delivers therapeutic cargo, and assesses the response to treatment. Uniformity and size control of CDs are critical steps and remain a challenge to attain stable properties and better performance for specific applications. However, several investigations have been done until now to achieve uniform and homogeneous CDs via either synthesis or post-treatment.

## Figures and Tables

**Table 1:** Data of drug loading and encapsulation efficiency of LNZ–BCDs nano-bioconjugates.

BCDs:LNZ	DEE (%)	DLE (%)
4:1	91.8 ± 1.3	20.7 ± 0.69
2:1	92.5 ± 1.4	21.1 ± 0.19
1:1	97.2 ± 1.0	22.5 ± 0.23
1:3	88.1 ± 1.9	19.8 ± 0.36
1:5	85.3 ± 1.1	18.6 ± 0.47

Results are expressed as mean ± SD.

**Table 2:** Parameters and *R*^2^ values of each kinetic model for estimating the release of LNZ from (1:1) LNZ–BCDs nano-bioconjugate.

	Kinetic Model					
	Zero Order	First Order	Second Order	Higuchi Diffusion	Hixon-Crowel	Baker-Lonsdal
*R* ^2^	0.804 *K*_0_ = 0.02714	0.956 *K*_1_ = 0.00094	0.985 *K*_2_ = 0.00005	0.915 *K_P_* = 1.872038	0.912 *K_β_* = 0.000917	0.957 *K_C_* = 0.00012

**Table 3:** Results of MIC of LNZ, BCDs nano-biocarrier, and LNZ−BCDs nano-bioconjugate against different bacterial strains.

Bacterial Strain	Minimum Inhibitory Concentration (MIC)		
	LNZ (µg/mL)	BCDs (mg/mL)	LNZ−BCDs (µg/mL)
*Staphylococcus aureus* (ATCC^®^ 25922)	4 ± 0.00 (S) *	11.25 ± 0.43	2 ± 0.00 (S)
Methicillin-resistant *Staphylococcus aureus* (MRSA)	4 ± 0.00 (S)	11.25 ± 0.43	0.25 ± 0.09 (S)

* S: sensitive.

**Table 4:** Effect of LNZ, BCDs nano-biocarrier, and LNZ–BCDs nano-bioconjugate on the percentage enzyme inhibition activity along with the IC_50_ (µg/mL) of various skin-related enzymes in comparison to their corresponding positive controls.

Test Sample	Anti-Elastase		Anti-Collagenase		Anti-Tyrosinase	
	% Inhibition	IC_50_ (µg/mL)	% Inhibition	IC_50_ (µg/mL)	% Inhibition	IC_50_ (µg/mL)
LNZ–BCDs	95.92 ± 2.21 ^a,b^	30.01 ± 1.13 ^a,b,c^	93.23 ± 1.65 ^a,c^	208.31 ± 3.08 ^a,b,c^	85.12 ± 1.50 ^a,b,c^	180.01 ± 2.12 ^a,c^
LNZ	88.00 ± 1.35	57.20 ± 2.88	65.05 ± 1.48	335.34 ± 2.09	65.00 ± 1.63	363.50 ± 2.08
BCDs	91.34 ± 1.47	45.50 ± 2.17	92.02 ± 1.25	253.66 ± 2.51	80.23 ± 1.41	180.40 ± 5.09
+ve control	92.52 ± 4.63	44.92 ± 1.71	79.82 ± 2.63	315.12 ± 2.21	76.52 ± 0.83	321.65 ± 3.41

Results are presented as mean ± standard deviation. ^a^, ^b^, and ^c^ represent significant difference from LNZ, BCDs nano-biocarrier, and +ve control, respectively.

## Data Availability

Data are available upon request.

## Supplementary Materials

The following supporting information can be downloaded at: https://www.mdpi.com/article/10.3390/pharmaceutics15010234/s1, Figure S1: Drug release kinetics of (a) Zero order, (b) First order, (c) Second order, (d) Higuchi diffusion, (e) Hixon-Crowel, and (f) Baker-Lonsdal kinetic models for the release of LNZ from (1:1) LNZ–BCDs nano-bioconjugate.
