# Supplementary material for: Green Synthesis of Highly Fluorescent Carbon Dots from Bovine Serum Albumin for Linezolid Drug Delivery as Potential Wound Healing Biomaterial: Bio-Synergistic Approach, Antibacterial Activity, and In Vitro and Ex Vivo Evaluation
Source: Pharmaceutics. 2023 Jan 10;15(1):234. doi: 10.3390/pharmaceutics15010234 (PMC9862409; doi:10.3390/pharmaceutics15010234)
Supplement: Supplementary file 1 [file pharmaceutics-15-00234-s001.zip › pharmaceutics-2143237-supplementary.pdf]

pharmaceutics

Supplementary Materials

Green Synthesis of Highly Fluorescent Carbon Dots from Bovine Serum Albumin for Linezolid Drug Delivery as Potential Wound Healing Biomaterial: Bio-Synergistic Approach, Antibacterial Activity, and In Vitro and Ex Vivo Evaluation

Dina Saeed Ghataty, Reham Ibrahim Amer, Mai A. Amer, Mohamed F. Abdel Rahman and Rehab Nabil Shamma

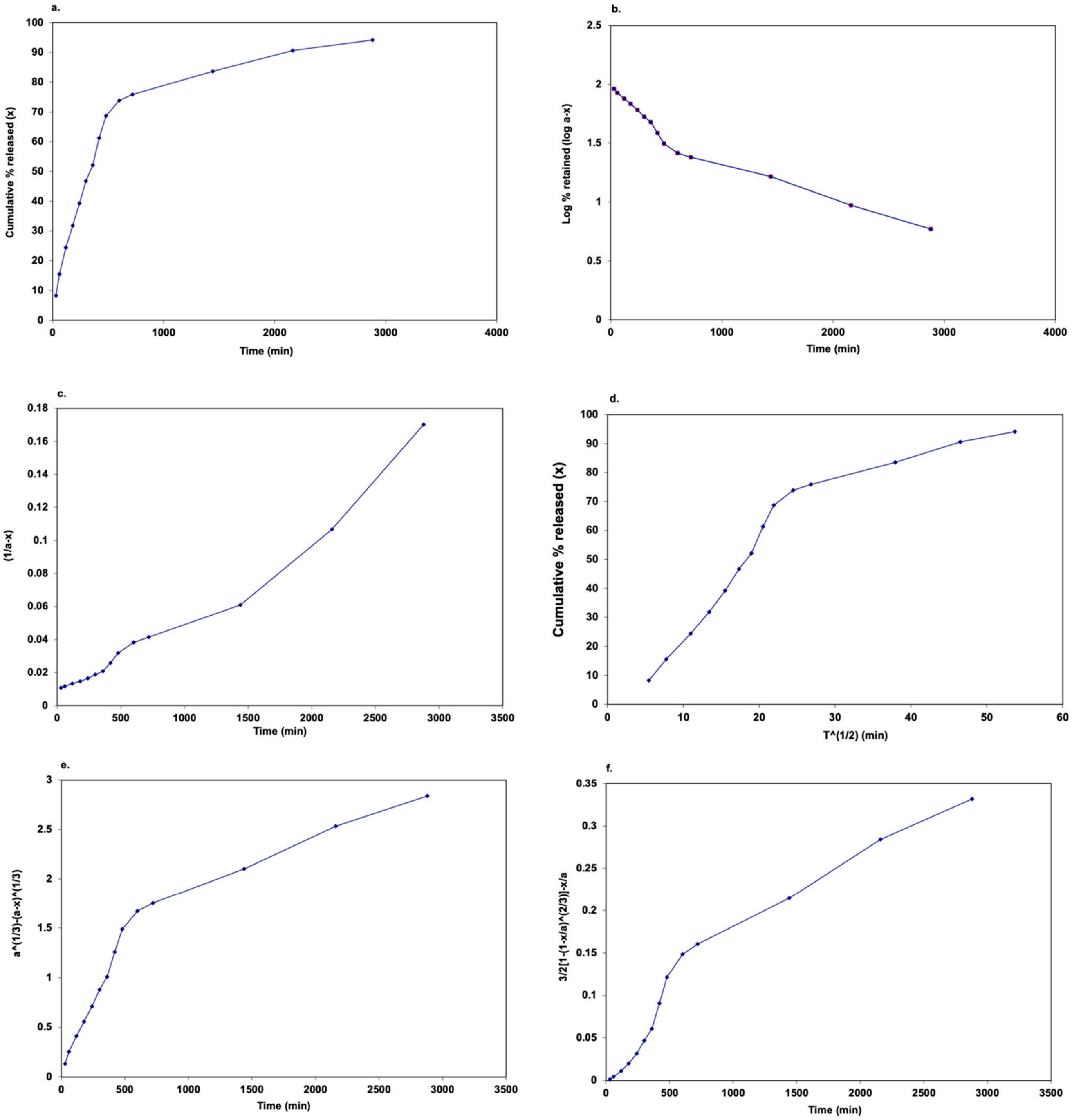

Figure S1. Drug release kinetics of (**a**) Zero order, (**b**) First order, (**c**) Second order, (**d**) Higuchi diffusion, (**e**) Hixon-Crowel, and (**f**) Baker-Lonsdal kinetic models for the release of LNZ from (1:1) LNZ–BCDs nano-bioconjugate.
